# Simultaneous Multiple-Stages Mpox Genital Lesions on the Same Site in a Traveler to Greece: A Case Report

**DOI:** 10.3390/vaccines11050901

**Published:** 2023-04-26

**Authors:** Anna Tagka, Styliani Geronikolou, Apostolos Evaggelopoulos, Sotiria Grigoropoulou, Dimitra Kavatha, Chryssoula Botsi, Aggeliki Papadopoulou, Kyriaki Tryfinopoulou, Antigoni Katsoulidou, Styliani Pappa, Anna Papa, Vasilios Paparizos, Electra Nicolaidou, Sotirios Tsiodras, Alexandros J. Stratigos

**Affiliations:** 1 1st Department of Dermatology and Venereology, Athens Medical School, “Andreas Syggros” Hospital for Skin and Venereal Diseases, National and Kapodistrian University, 15772 Athens, Greece; 2 Clinical Translational and Experimental Surgery Center, Biomedical Research Foundation of the Academy of Athens, 11527 Athens, Greece; 3 4th Department of Internal Medicine, Athens Medical School, National and Kapodistrian University, 11527 Athens, Greece; 4 Central Public Health Laboratory, National Public Health Organization, 15123 Athens, Greece; 5 Department of Microbiology, Medical School, Aristotle University of Thessaloniki, 54124 Thessaloniki, Greece

**Keywords:** MPXV, mpox, travel patient, respiratory tract symptoms, case report, multiple-stages lesions

## Abstract

A 47-year-old Caucasian traveller from an mpox (formerly monkeypox and also best suited abbreviated MPX)-endemic country was referred for a skin rash, of recent onset, confined to the genital area. The rash consisted of erythematous umbilicated papules, vesicles and pustules with a characteristic white ring. The lesions were observed simultaneously at different stages of progression on the same anatomical site, a clinical presentation that is not encountered frequently. The patient was febrile, fatigued and had blood-tinged cough. The clinical suspicion of mpox was raised, and the initial real-time PCR identified a non-variola orthopox virus, which was confirmed at the National Reference Laboratory to belong to the West African clade.

## 1. Introduction

“Case reports are the starting point to medical science” and often point to new epidemics [1]. Mpox is caused by a double-stranded DNA virus that belongs to the *Poxviridae* family (genus Orthopoxvirus): MPX virus (MPXV)] [2,3]. Clusters of the disease over all World Health Organisation (WHO) surveillance regions revealed a new epidemic, resulting in the declaration of mpox as a global health emergency οn 23 July 2022 [4]. Thus, vigilance (patient isolation, contact tracing and personal protection measures) in combatting transmission of this virus was considered urgent. Up until then, mpox infection was considered a zoonotic infection that was rarely observed in local African clusters. According to the European Centre for Disease Prevention and Control (ECDC), as of 4 April 2023, 21,212 cases have been identified in the 29 EU countries out of 25,874 cases identified by ECDC and the WHO Regional Office for Europe through The European Surveillance System (TESSy) for the 41 included European countries [5]. In Europe, the first case was retrospectively identified through a residual specimen, dated 7 March 2022. The first symptoms onset was recorded on 17 April 2022 [5].

Prior to the recent outbreak, the disease was typically diagnosed in individuals who had travelled from endemic countries, as well as in a small number of household contacts, and was less commonly diagnosed in healthcare workers [6,7]. To date, only four occupational cases have been identified within the European territories concerning healthcare workers that had taken no or flawed protection measures [5]. Globally, as of 8 April 2023, the WHO reports 86,838 laboratory-confirmed cases plus 1050 probable cases and 112 deaths in 110 countries that report mpox. The risk is reported as moderate globally, as well as in Europe, the African region, the South-Eastern region and the regions of the Americas. Concerning the South-East Asia Region and the Western Pacific Region, the risk has been estimated as low [8]. Notably, the frequency of reporting new cases is declining, at present: “In the most recent week of full reporting, 10 countries reported an increase in the weekly number of cases, with the highest increase reported in Panama. In the past 21 days, 26 countries have reported cases” [8].

The current global epidemic has exhibited differential prevalence in terms of the age and gender structure of the affected populations i.e., younger males, with approximately half of the cases occurring in people around 30 years old. The majority of patients exhibit a clinical presentation characterised by atypical lesions in the anal and genital area as well as a rash that tends to spare the face and extremities. This is typically accompanied by fever, followed by inguinal lymphadenopathy as well as myalgia and fatigue [9,10]. Risk factors associated with acquisition of the disease include being a man engaging in high-risk sexual activities with other men (MSM), including condomless sex, frequent intercourse with unknown partners, as well as previous history of infection with Human Immunodeficiency Virus (HIV) and any other sexually transmitted infections [9,10,11,12,13,14].

It is established that neutralising antibodies as a result of a previous infection or vaccination are key players in the viral infections control. As reported by Pelegrin et al., vaccine-like neutralising antibodies may trigger cellular as well as humoral immune responses [15]. As the post exposure vaccina virus-based smallpox vaccine is not considered a harmless one, other treating options were investigated for orthopox viruses. Currently, there is no specific treatment for this form of pox. The smallpox treating options consist of the available quiver of mpox care providers. Tecovirimat is a drug approved by the Food and Drug Administration (FDA) to treat smallpox in adults and children. Both the Centers for Disease Control and Prevention (CDC) and ECDC suggests that it should be given to severe cases or to those at high risk groups (such as those having weak immune system due to prior infections or chronic diseases or immunosuppressive drug intake). The same drug may serve as prophylactic for short-term severe symptoms such as pain, swelling, scarring. Yet, unravelling COP-C3L gene (a complementary control protein) variants might assist future mpox specific drug treatment [10].

In the current manuscript, we describe a unique clinical presentation of mpox not previously described, where the patient presented with skin lesions at different stages of progression on the same anatomical site. This manuscript has been constructed in accordance with CARE guidelines and the relevant declaration may be found as Supplementary Materials (CARE checklist).

## 2. Case Report

On 11 June 2022, a 47-year-old Caucasian male patient came to the emergency unit of A. Syggros Hospital in Athens, Greece, seeking care for skin lesions confined to the genital area that had developed over the past 96 h. The exam identified a spreading rash of pink-to-red spots located mainly in the pubic area and the penis. The lesions consisted mainly of papules, vesicles and pustules, all simultaneously present at the site of rash, associated with local pain. The initial umbilication has progressed to a small necrotic crust with depression in the central area. Schemes follow the same pattern in both sites of genital region. Several systemic features were reported including high fever, arthralgias, myalgias and fatigue. The patient had a medical history of two hospital admissions for pneumonia over the past two years. He also reported himself as an MSM.

**Figure 1:**
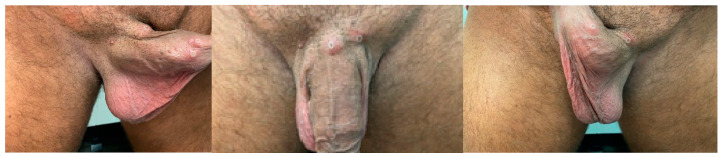
Multiple lesions (evolving from papules, vesicles and to pustular stage) located in pubic area and the penis in different stages of progression. The initial umbilication has progressed to a small necrotic crust with depression in the central area. Schemes follow the same pattern in both sites of genital region.

During clinical examination, he manifested painful regional bilateral inguinal lymphadenopathy, along with 4–5 mm umbilicated or intact vesicles and papules on the penis base and shaft resembling mpox. Based on the patient’s travel history (he had travelled from Spain, a mpox endemic country at that time) and his sexual history, mpox was suspected, likely acquired through human contact. Most interestingly, multiple stages of genital lesions were simultaneously present in the same site (Figure 1). To our knowledge, this is an uncommon presentation, not previously described in the literature [16,17,18,19].

His vital signs included fever (T = 39.1 °C), a blood pressure of 130/85 mmHg, a heart rate of 100 bpm, a respiratory rate of 20 breaths per min and an oxygen saturation (SpO_2_) of 96%.

At admission, the patient exhibited a non-productive cough, with occasionally blood tinged sputum. A rapid test for SARS-CoV-2 was negative.

**Figure 2:**
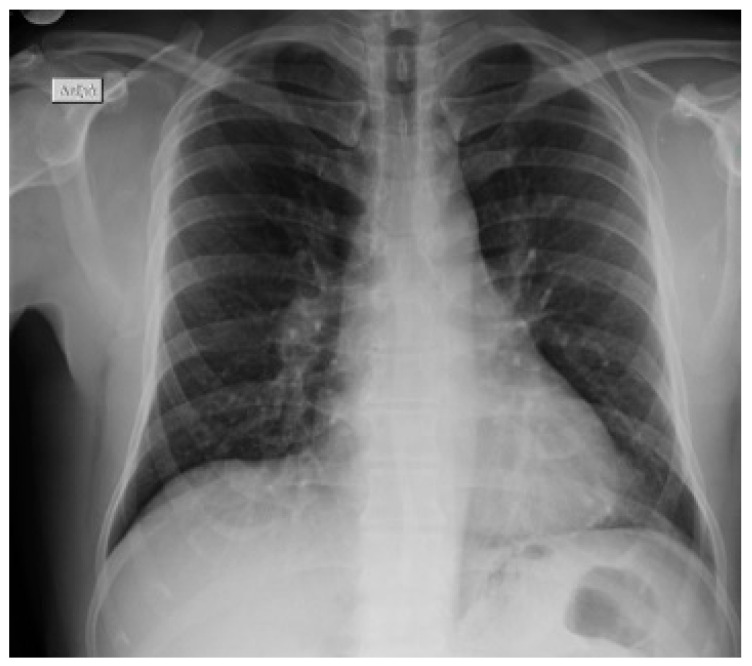
Patient’ s X-ray after he was admitted to the emergency department.

He was subjected to isolation, pending further investigation. Lung auscultation demonstrated a mildly reduced intensity of breath sounds mainly at the bases. The blood tests showed normal white blood cells (5.690/μL, 74.8% neutrophils and 14.1% lymphocytes), increased erythrocyte sedimentation rate (ESR 35 mm per hour) and normal biochemical testing (glucose, urea, creatinine, electrolytes and liver function tests). The arterial blood gas sample analysis showed a respiratory alkalosis with a pH 7.461, a pO_2_ of 82.8 mmHg, a pCO_2_ of 25 mmHg, an O_2_ saturation of 97% and a HCO_3^−^_ of 18 mmol/L, while the lactate level was of 0.8 mmol/L). The chest X-ray depicted an increase in overall lung markings and elevation of the right hemidiaphragm with no consolidation or other findings (Figure 2).

Due to fever persistence and the associated respiratory findings, the patient was transferred to the 4th University Department of Internal Medicine, at Attikon General Hospital for further management on day 2 after his initial admission. There, he was examined for HIV and been found negative.

**Figure 3:**
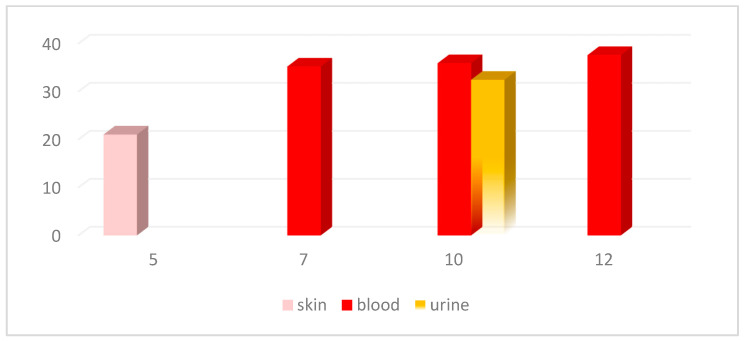
Cycle threshold (Ct) values in MPXV-specific real-time PCR (Y axis) in various sample types according to the day of patient’s illness (X axis). On day 12, the urine specimen was negative for MPXV.

Specimens from skin lesions (skin swabs) were sent to the Central Laboratory of Public Health in Athens, Greece, and non-variola orthopox virus was detected by real time PCR. The DNA was sent to the reference laboratory at Aristotle University of Thessaloniki, Greece, where mpox was confirmed by applying a MPXV-specific real-time PCR following the protocol from CDC (Test Procedure: Non-variola Orthopoxvirus Generic Real-Time PCR Test (https://www.cdc.gov/poxvirus/mpox/pdf/PCR-Diagnostic-Protocol-508.pdf, accessed on 2 June 2022) the cycle threshold (Ct) value was 21.04 (Figure 3). An additional PCR was applied, using the OPS3 and OPAs4 primers and the protocol described by Panning et al. (2004) [20]. The 270-bp PCR product was Sanger sequenced; Blast analysis showed that the obtained sequence was identical to sequences belonging to the West African clade of MPXV, which has driven the outbreak in Europe.

Multiple biological samples including skin, blood and urine samples were obtained with patient consent to evaluate progression of viral load on days 5, 7, 10 and 12 after symptoms onset (Table 1, Figure 1). The samples were analyzed at the Academic Reference Laboratory in the Aristotle University of Thessaloniki. DNA was extracted from all sample types using the QIAamp DNA Mini Kit (QIAGEN, Hilden, Germany). The above mentioned MPXV-specific real-time PCR was applied, and Ct values were taken, as described in Table 1, and Figure 3.

The patient was closely followed for any further clinical deterioration at the isolation unit of the 4th Department of Medicine and received symptomatic interventions including pain relief with paracetamol and topical lidocaine. He gradually improved without the need of systemic antiviral therapy. He was discharged after one week and was advised to remain isolated until all lesional scabs had resolved. The patient’s contacts were advised to continue their daily activities as long as they remained asymptomatic; self-monitoring for symptoms for 21 days after their last exposure was strongly advised as well as immediate medical attention should any symptom developed. Although the efficacy of post exposure vaccination has been established during previous outbreaks, none of the orthopox virus vaccines were ntionally available at that time.

## 3. Discussion

The diagnosis of mpox is based on history, clinical symptoms, examination and laboratory tests. Confirmatory or differential diagnosis is critical in order to exclude other possible infectious diseases. The first mpox case in Greece was reported on 8 June 2022 [13]. The case described in the present study was the second diagnosed mpox case in the country. It was a case imported from Spain, a country that first reported human-to-human transmission of the Western African clade of the disease beyond Africa. Spain reported the first case on 18 May 2022. This country has reported 7805 in the governmental official cite and 7549 in the ECDC. This patient presented multiple erythematous umbilicated papules, vesicles and pustules with a characteristic white ring, located in the pubic area and the penis at different stages of progression. The initial umbilication progressed to a small necrotic crust in central area as previously described [12,21,22].

Lesion swabs are the standard sample type for mpox infection laboratory diagnosis by PCR. Additional sample types, such as plasma, saliva, oropharyngeal swabs and urine can be also used, although with lower sensitivity. Testing specimens from multiple sites may improve the sensitivity and reduce false-negative test results [23,24]. As expected, the lowest Ct value was seen in the initial skin swab, while increasing Ct values (that means decreasing viral load) were seen in the blood and urine samples.

As for 13 February 2023, the WHO has confirmed 86,838 laboratory confirmed plus 1050 probable cases and 112 deaths in 110 countries [25]. Notably, the outbreak is subsiding and there were no reported cases or deaths in the Eastern Mediterranean (including Greece) during February 2023. Of the available global case data, 96.5% (73,345/76,044) of the affected individuals were men, with 84.2% (26,967/32,043 being bisexual or MSM, with a median age of 34 years (interquartile range: 29–41 years). Only 1.1% (918/81,806) of cases involved children aged 0–17 years, with 267 (0.3%) occurring in children aged 0–4 years. The majority of cases involving children were reported in the Region of the Americas (667/918; 73%), where children accounted for 1.2% (667/55,638) of all reported cases [25].

Typically, during this outbreak, patients presented with fatigue, fever, exanthema, anal and genital lesions as well as inguinal lymphadenopathy [25]. Breggazi et al. summarised that in six clusters in European countries accounting for 124 cases (54.3% male gender), the clinical image included inguinal lymphadenopathy in (45.7%) and rash (40%), fatigue (22.9%), myalgia (17.4%), headache (25.7%) and anogenital lesions (31.4%) of the cases [9].

Mild disease presentations are more frequently encountered; however, the spectrum includes complicated severe disease with deep tissue abscess, sepsis or even respiratory disease [26,27,28,29,30,31,32].

The unique finding in the presented case is the presence of skin lesions at differential stages of progression at the same anatomical area 96 h after symptoms onset, a characteristic of other viral exanthemas such as the one by Varicella zoster. This uncommon picture has not been previously described, to the best of our knowledge, and raises the importance of rapid molecular testing in suspect mpox cases with strong epidemiological history.

In addition, respiratory involvement similar to our case has been previously described with concomitant viral shedding in reports from the United Kingdom (UK) [33]. These clinical findings led to extended isolation in our case in Greece, as was the case in previous cases reports from the UK and Belgium. A neutralising monoclonal antibody targeting the virus can promote virus entry into cells by the Fc region of the antibody bound to the Fc receptor (FcR) on cells; this is correlated with disease progression and poor outcomes of patients with mpox. The pathogenesis of such cases involves NK cell function impairment (impaired IFN-γ and TNF-α secretion via inhibition of the expression of chemokines (CCR5, CXCR3 and CCR6)), lymphopenia, a cytokine storm (increase in IL-4, IL-6, IL-5, IL-8 and IL-10 and attenuation of Th1-associated cytokines, such as IFN-α, IFN-γ, TNF-α, IL-2 and IL-12.7), increased blood monocytes and granulocytes (including basophil, eosinophil, neutrophil and monocyte), immune evasion (by suppressing the antiviral type 1 IFN responses), inhibition of the host complement system and antibody-dependent enhancement [34].

Human-to-human transmission of mpox occurs mainly through physical contact with infected skin and body fluids. Another mode of transmission is exposure to respiratory droplets and fomites [11,35]. A rapid replication at the inoculation site occurs which spreads to regional lymph nodes. The rash might appear on the genitals (penis, testicles, labia, vagina) or anus. Additionally, lesions could be located in other areas such as the hands, feet, face, mouth and chest.

A mild rash was present in the first imported case of the infection in Greece from Portugal—a country where the disease was already prevalent [13]. The favourable clinical outcome in that patient was attributed to his past vaccination history and immunisation for smallpox. On the contrary, the second diagnosed case (the one presented in this paper) reported no previous vaccination for smallpox nor HIV co-infection, which may explain the more diverse and systemic manifestations observed in this patient. HIV infection may affect the clinical course of the disease [14].

It has been suggested that vaccination against smallpox provides 85% cross-protection against MPXV if given before exposure [36,37]. As small pox had been considered eradicated for decades, many countries opted not to continue smallpox massive vaccination. This could partly influence the population’s herd immunity and, in part, be responsible for the current mpox outbreak in humans [14]. Nevertheless, in a multi-centre, prospective, observational cohort study in three sexual health clinics in Spain, 32 individuals acquired MPX despite smallpox vaccination in their childhood. Further investigation and better understanding of the smallpox vaccination effectiveness on mpox prevalence appears to be critical [38]. Since the level of protection and duration of immunity is uncertain, and individuals with prior smallpox vaccination can still be susceptible to the virus, the CDC recommends vaccination with the MPX vaccine for individuals at high risk of exposure to the virus.

From the three generations of vaccines against orthopox viruses available, the second generation (ACAM2000 in the United States of America (USA)) is not recommended for patients with atopic dermatitis or immunosuppressed patients (i.e., those suffering from acquired immunodeficiency syndrome (AIDS)). The modified vaccinia Ankara, Bavarian Nordic, a third generation vaccine, referred to as JYNNEOS in the USA, IMVAMUNE in Canada and IMVANEX in Europe, might provide better protection to immunocompromised subjects [39,40,41]. An alternative option for such vulnerable populations is the third-generation recently modified LC16 ma8—an attenuated vaccinia virus derived from the Lister (Elstree) strain, which was approved in Japan in 1975 [41]. If given after infection, the vaccines provide lower protection. Concerning the prophylaxis of the at-risk population (i.e., contacts), the efficacy of such a measure depends on the authorities’ reaction level [34].

People who are vaccinated should continue to avoid skin-to-skin contact with someone who has active mpox infection. Self-isolation for mpox patients is proposed by national guidance.

Mpox is no longer considered to be ‘‘just another neglected disease”. Careful evaluation of confirmed cases with uncommon presentations increases our understanding and early recognition of the disease and improves the effectiveness of measures including isolation, tracing contacts, antiviral treatment and vaccine interventions. In this context, beside reports of case series, individual cases studies with atypical clinical presentations are of additive value for early recognition and response.

To this end, in terms of future clinical diagnosis, simultaneous multiple-stages skin lesions on the same site (even if observed shortly after symptoms onset) may be a sign of systemic infection.

## Figures and Tables

**Table 1:** Cycle threshold (Ct) values in MPXV-specific real-time PCR in various sample types according to the day of patient’s illness.

Sample Type	Date of Sampling	Day of Illness	Ct in MPXV Real Time PCR
Skin swab	11 June 2022	5th	21.04
Blood	12 June 2022	7th	35.21
Blood	15 June 2022	10th	35.91
Urine	15 June 2022	10th	32.44
Blood	17 June 2022	12th	37.59
Urine	17.June 2022	12th	Negative

## Data Availability

All data available are included in the text. Personal data are not available due to privacy restrictions.

## Supplementary Materials

The following supporting information can be downloaded at: https://www.mdpi.com/article/10.3390/vaccines11050901/s1. CARE checklist.

## Abbreviations

HIV: human immunodeficiency virus, MPXV: monkeypox virus, MSM: men that have sex with men, RT-PCR: real time reverse transcription PCR, WHO: World Health Organisation, UK: United Kingdom, EU: European Union; CDC: Centers for diseases and prevention; AIDS: acquired immunodeficiency syndrome.
